# Efficient itaconic acid production from glycerol with *Ustilago vetiveriae* TZ1

**DOI:** 10.1186/s13068-017-0809-x

**Published:** 2017-05-19

**Authors:** Thiemo Zambanini, Hamed Hosseinpour Tehrani, Elena Geiser, Dorothee Merker, Sarah Schleese, Judith Krabbe, Joerg M. Buescher, Guido Meurer, Nick Wierckx, Lars M. Blank

**Affiliations:** 1 Institute of Applied Microbiology-iAMB, Aachen Biology and Biotechnology-ABBt, RWTH Aachen University, Worringerweg 1, 52074 Aachen, Germany; 2 BRAIN AG, Darmstädter Straße 34, 64673 Zwingenberg, Germany

**Keywords:** Adaptive laboratory evolution, Fed-batch cultivation, Glycerol, Itaconate, *Ustilago vetiveriae*

## Abstract

**Background:**

The family of Ustilaginaceae is known for their capability to naturally produce industrially valuable chemicals from different carbon sources. Recently, several Ustilaginaceae were reported to produce organic acids from glycerol, which is the main side stream in biodiesel production.

**Results:**

In this study, we present *Ustilago* *vetiveriae* as new production organism for itaconate synthesis from glycerol. In a screening of 126 Ustilaginaceae, this organism reached one of the highest titers for itaconate combined with a high-glycerol uptake rate. By adaptive laboratory evolution, the production characteristics of this strain could be improved. Further medium optimization with the best single colony, *U.* *vetiveriae* TZ1, in 24-deep well plates resulted in a maximal itaconate titer of 34.7 ± 2.5 g L^−1^ produced at a rate of 0.09 ± 0.01 g L^−1^ h^−1^ from 196 g L^−1^ glycerol. Simultaneously, this strain produced 46.2 ± 1.4 g L^−1^ malate at a rate of 0.12 ± 0.00 g L^−1^ h^−1^. Due to product inhibition, the itaconate titer in NaOH-titrated bioreactor cultivations was lower (24 g L^−1^). Notably, an acidic pH value of 5.5 resulted in decreased itaconate production, however, completely abolishing malate production. Overexpression of *ria1* or *mtt1*, encoding a transcriptional regulator and mitochondrial transporter, respectively, from the itaconate cluster of *U.* *maydis* resulted in a 2.0-fold (*ria1)* and 1.5-fold (*mtt1*) higher itaconate titer in comparison to the wild-type strain, simultaneously reducing malate production by 75 and 41%, respectively.

**Conclusions:**

The observed production properties of *U.* *vetiveriae* TZ1 make this strain a promising candidate for microbial itaconate production. The outcome of the overexpression experiments, which resulted in reduced malate production in favor of an increased itaconate titer, clearly strengthens its potential for industrial itaconate production from glycerol as major side stream of biodiesel production.

## Background

The switch from a mainly petroleum to a sustainable bioeconomy has become omnipresent over the last years. Consequently, research is focusing on the development of biotechnological production processes, resulting in biochemicals able to compensate for petrochemicals. One group of these chemicals is organic acids, such as succinate, malate, or itaconate.

Especially the C5-dicarboxylic acid, itaconate, has gained great interest, due to a broad range of possible applications in different industries and technologies, such as in carbon fibers, rubber, anti-scaling polymers in water treatment, cement additives, surface active agents, plastics, and dye intermediates [1–3]. Additionally, it can be converted into different value-added molecules, due to its multiple functional groups [1] or be used for self-polymerization to poly-itaconate, which has the potential to replace a broad range of different polymers [4–6]. In 2004, itaconate was announced one of the top twelve building block chemicals, to be produced from renewable biomass, by the U.S. Department of Energy [1]. Even though the contemporary market for itaconate is rather small with about 41 kt a^−1^ in 2013, corresponding to a market value of approximately US$ 74.5 million, it is predicted to reach US$ 570 million by 2020 [7]. This huge increase in the expected market volume is based on the possibility to substitute existing chemicals, if an improved production process can be developed that would lower the price for itaconate.

Chemical synthesis of itaconate was first reported in 1836 [8–10] and in 1931, *Aspergillus* *itaconicus* was the first organism to be found to produce itaconate [11]. In contrast to many other chemicals, contemporary itaconate production is completely achieved by biotechnological processes [2]. These processes mainly rely on *A.* *terreus* strains. The first *A.* *terreus* strain producing itaconate was discovered in 1939 [12] and since then, the use of this organism has been investigated and improved intensively for the production of itaconic acid [13–15]. Over the years, many different organisms have been found to produce itaconate, including several species of *Pseudozyma* [16, 17], *Ustilago* [18–21], and different *Candida* [22] and *Rhodotorula* [23] species.

Many of these production strains are a member of the family of Ustilaginaceae, which is a promising fungal family for biotechnological applications [24–26]. Recently, the itaconate production pathway in *U.* *maydis* has been clarified, allowing for targeted metabolic engineering of itaconate production in this host [27, 28].

The family of Ustilaginaceae is generally known for combining natural production of different industrially relevant products, such as organic acids, polyols, and lipids from a broad range of substrates, with favorable characteristics for biotechnological processes, such as a yeast-like morphology, insensitivity to medium impurities and tolerance to high product titers [4, 19–21, 24, 25, 29–35]. Especially, the broad substrate range attracted interest in this group of organisms. As plant pathogens, Ustilaginaceae are able to degrade a broad range of polymers from biomass, such as cellulose, hemicellulose, or xylan [36–39]. Recently, malate production from biodiesel-derived glycerol has been demonstrated with *Ustilago* *trichophora* TZ1 [40–42]. The use of glycerol as substrate for microbial conversion has been discussed frequently over the last years. In a follow-up study of the landmark 2004 DOE report [1], glycerol is still considered as one of the 10 most promising building blocks to be produced [43]. However, worldwide increasing biodiesel production has resulted in a huge side stream of (crude) glycerol, which makes up 10% (w/v) of the total production. With 123 million tons of biodiesel per year predicted for 2016 [44], 19 million tons of crude glycerol will flood the market, further lowering the price, while simultaneously decreasing the profit margin for the biodiesel production process itself. Consequently, valorization of this huge waste-stream has been discussed intensively, resulting in several microbial production processes for different products starting from glycerol [45, 46].

Here we present *U.* *vetiveriae* TZ1 as promising production organism for organic acids from glycerol, reaching high total acid titers with itaconate and malate as the main products. Further, we demonstrate that by single overexpression of two different genes, the acid production profile can be drastically influenced in favor of itaconate.

## Results and discussion

### Submersed cultivation reveals *U.* *vetiveriae* as a promising itaconate producer

**Fig. 1:**
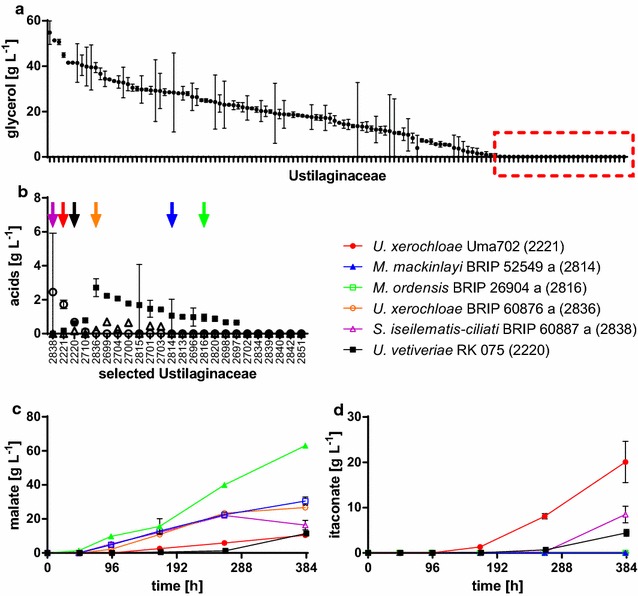
Screening for the production of organic acids from glycerol. **a** Rank-ordered glycerol concentration after 355 h of cultivation in 24-deep well plates containing MTM with 50 g L^−1^ glycerol, 0.8 g L^−1^ NH_4_Cl, and 100 g L^−1^ CaCO_3_ for 126 different Ustilaginaceae. The glycerol concentrations and strain numbers are indicated in Table 3. Strains with high-glycerol consumption (indicated by the *red box*) were re-screened for acid production. **b** Rank-ordered itaconate concentration (*open circles*), malate concentration (*closed squares*), and succinate concentration (*open triangles*) after 383 h of cultivation in 24-deep well plates containing MTM with 100 g L^−1^ glycerol, 0.8 g L^−1^ NH_4_Cl, and 100 g L^−1^ CaCO_3_ for 24 selected Ustilaginaceae. Strains with good production (indicated by *arrows*) were evaluated in detail. **c** Malate concentration and **d** itaconate concentration for different Ustilaginaceae cultivated in shake flasks containing MTM with 200 g L^−1^ glycerol, 0.8 g L^−1^ NH_4_Cl, and 100 g L^−1^ CaCO_3_. *Error bars* indicate deviation from the mean (*n* = 2)

Recently, we reported on an *U.* *trichophora* strain, which was found in a broad screening of Ustilaginaceae, to naturally produce malate from glycerol [40]. The primary screening in this study was performed on agar plates with a pH indicator, only resulting in a qualitative indication of growth and semi-quantitative indication concerning total acid production. Due to the generally high malate production of many Ustilaginaceae [21], this method is less suited for finding producers of other organic acids such as itaconate. Consequently, we performed a complete screening of 126 Ustilaginaceae cultivated in 24-deep well plate liquid cultures [47] containing mTM with 50 g L^−1^ glycerol, 0.8 g L^−1^ NH_4_Cl, and 100 g L^−1^ CaCO_3_. After 355 h, the culture supernatants were initially evaluated for glycerol uptake (Fig. 1a) and strains with the highest glycerol uptake rate were selected for further analysis.

As reported previously for Ustilaginaceae [21, 40], a broad phenotypic distribution was observed. Some strains consumed all carbon, while others did not grow at all on glycerol (Fig. 1a). Although organic acid production was observed with a broad diversity, titers were generally low due to the low concentration of glycerol used. Hence, we chose the 24 strains with fastest glycerol uptake (indicated by the red box in Fig. 1a), to investigate in a second 24-deep well plate screening with a higher initial glycerol concentration of 100 g L^−1^ (Fig. 1b). From this screening, the six strains with best itaconate and malate production properties (Fig. 1b) and highest glycerol uptake (indicated by arrows in Fig. 1b) were chosen and cultivated in shake flasks containing 200 g L^−1^ glycerol. Only three of these strains, *U.* *vetiveriae*, *U.* *xerochloae*, and *Sporisorium iseilematis*-*ciliati* were able to produce itaconate, reaching a titer of 4.4 ± 0.8, 20.1 ± 4.6, and 8.5 ± 1.8 g L^−1^, respectively, while all strains produced malate with titers between 10.5 ± 0.7 and 63.1 ± 0.3 g L^−1^. Even though production rates and titers for itaconate are lower than for *A.* *terreus* [48] and *P.* *antarctica* [16] on glucose, they are rather high for wild-type strains, given the applied conditions, leaving space for improvement.

### Adaptive laboratory evolution improves growth and acid production on glycerol

**Fig. 2:**
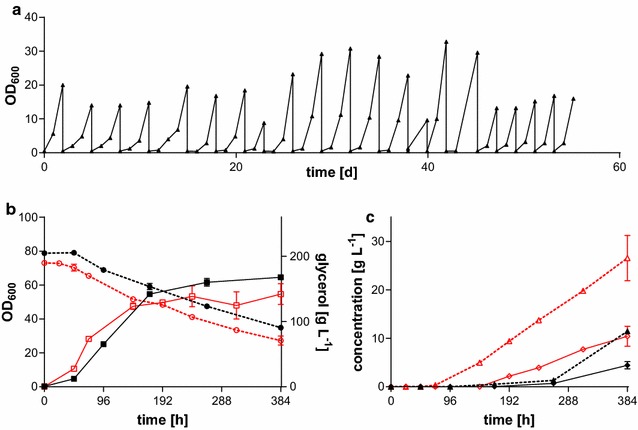
Adaptive laboratory evolution of *U.* *vetiveriae* RK075. **a** ALE of *U.* *vetiveriae* on glycerol as sole carbon source. A single representative culture is shown. **b** Comparison of growth (*squares*, *solid lines*) and glycerol consumption (*circles*, *dashed lines*) and **c** itaconate (*diamonds*, *solid lines*) and malate (*triangles*, *dashed lines*) production for *U.* *vetiveriae* RK075 (*black*, *closed symbols*) and *U.* *vetiveriae* TZ1 (*red*, *open symbols*) on glycerol.* Error bars* indicate deviation from the mean (*n* = 2)

Adaptive laboratory evolution (ALE) is known to be suitable for the improvement of specific microbial characteristics by adaptation to the chosen environmental conditions and selection of beneficial mutations [49–51]. Recently, we were able to improve malate production, growth rate, and glycerol uptake in *U.* *trichophora* by ALE [40]. Here we used the same re-inoculation scheme in shake flasks applying faster growth rate and consequently higher glycerol uptake rates as selection pressure for all six strains from the last screening in duplicates. While growth rate could not be increased except for *U.* *vetiveriae*, glycerol uptake was improved for all strains. Additionally, malate production was improved for all strains (Table 1). Figure 2a shows the results for *U.* *vetiveriae* RK075. Although the maximum glycerol uptake rate was not improved for this strain, the initial glycerol consumption was significantly improved by ALE, indicating an increased growth rate before the onset of nitrogen limitation.

**Table 1:** Outcome of the ALE

	Growth (h^−1^)	Glycerol rate (g L^−1^ h^−1^)	Itaconate titer (g L^−1^)	Itaconate rate (g L^−1^ h^−1^)	Malate titer (g L^−1^)	Malate rate (g L^−1^ h^−1^)
*U. vetiveriae* RK 075	0.05 ± 0.00 (0.07 ± 0.00)	0.30 ± 0.01 (0.31 ± 0.03)	4.4 ± 0.8 (10.4 ± 2.1)	0.01 ± 0.00 (0.03 ± 0.01)	11.5 ± 0.5 (26.6 ± 4.7)	0.03 ± 0.00 (0.07 ± 0.01)
*U. xerochloae* UMa702	0.05 ± 0.00 (0.05 ± 0.00)	0.35 ± 0.0 (0.63 ± 0.00)	20.1 ± 4.6 (0.0 ± 0.0)	0.05 ± 0.01 (0.00 ± 0.00)	10.5 ± 0.7 (81.2 ± 8.4)	0.03 ± 0.00 (0.21 ± 0.02)
*Macalpinomyces mackinlayi* BRIP 52549 a	0.05 ± 0.00 (0.05 ± 0.00)	0.38 ± 0.00 (0.64 ± 0.01)	0.0 ± 0.0 (0.0 ± 0.0)	0.00 ± 0.00 (0.00 ± 0.00)	63.1 ± 0.3 (35.5 ± 0.3)	0.17 ± 0.00 (0.09 ± 0.00)
*M. ordensis* BRIP 26904 a	0.04 ± 0.00 (0.04 ± 0.00)	0.30 ± 0.01 (0.62 ± 0.01)	0.0 ± 0.0 (0.0 ± 0.0)	0.00 ± 0.00 (0.00 ± 0.00)	30.6 ± 2.3 (79.6 ± 2.5)	0.08 ± 0.01 (0.21 ± 0.01)
*U. xerochloae* BRIP 60876 a	0.04 ± 0.00 (0.04 ± 0.00)	0.26 ± 0.01 (0.65 ± 0.00)	0.0 ± 0.0 (0.0 ± 0.0)	0.00 ± 0.00 (0.00 ± 0.00)	26.8 ± 1.4 (37.3 ± 2.1)	0.07 ± 0.00 (0.10 ± 0.01)
*S. iseilematis*-*ciliati* BRIP 60887 a	0.04 ± 0.00 (0.04 ± 0.00)	0.38 ± 0.00 (0.50 ± 0.00)	8.5 ± 1.8 (0.0 ± 0.0)	0.02 ± 0.01 (0.00 ± 0.00)	16.4 ± 2.8 (1.4 ± 0.4)	0.04 ± 0.01 (0.00 ± 0.00)

Comparison of growth rate, glycerol uptake rate and organic acid (itaconate, malate) titer and production rate before and after ALE. Values in brackets correlate to values after ALE. ±values indicate deviation from the mean (*n* = 2)

Since the malate titers stayed below the titer reached with the previously published *U.* *trichophora* TZ1 [40, 41, 52] or *A.* *oryzae* [53, 54], we focused on the strains producing itaconic acid. However, none of the initially identified itaconate producers produced itaconate after the 25th re-inoculation. Investigation of the cultures after 21 rounds of re-inoculation revealed that only *U.* *vetiveriae* was still able to produce itaconic acid (Table 1). Apparently, itaconate production is detrimental to the fitness of these strains under the applied ALE conditions, leading to lower or completely abolished production after prolonged ALE. The putative natural functions of itaconate include the competition with other microbes by a drop in pH and the liberation of micronutrients through chelating properties [27], both not required under these laboratory conditions. However, itaconate production is not expected to occur as long as a nitrogen source is present. Possibly, an altered ALE strategy with elevated ammonium levels and re-inoculation at lower cell densities may avoid the loss of itaconate production during ALE. Another possibility would be the investigation of itaconic acid production for all evolved strains after each re-inoculation. Even though *U.* *vetiveriae* stopped itaconate production after the 25th re-inoculation during ALE, it was still able to produce considerable amounts of itaconate after 21 re-inoculations. The best single colony (*U.* *vetiveriae* TZ1) isolated from the 21st re-inoculation, which corresponds to about 105 generations, produced 10.4 ± 2.1 g L^−1^ itaconate within 384 h at a production rate of 0.03 ± 0.01 g L^−1^ h^−1^ (Fig. 2c). In comparison, the reference strain (before ALE) produced 4.4 ± 0.8 g L^−1^ at a rate of 0.01 ± 0.00 g L^−1^ h^−1^ (Fig. 2c). Since the supplied amount of nitrogen was the same in both cultures and the final optical density was in the same range, this increase can actually be attributed to a higher specific production rate (g_malate_ g_biomass_^−1^ h^−1^). Additionally, malate production in this strain was improved, reaching 26.6 ± 4.7 g L^−1^ at a rate of 0.07 ± 0.01 g L^−1^ h^−1^, whereas the reference produced 11.5 ± 0.5 g L^−1^ at a rate of 0.03 ± 0.00 g L^−1^ h^−1^. Although the itaconic acid titer of *U.* *xerochloae* in the initial screening was higher, we focused on the evolved *U.* *vetiveriae* strain for further investigation because morphological and physiological characteristics of *U. xerochloae* (e.g., filamentous growth) interfered with downstream analytics and reproducibility.

**Fig. 3:**
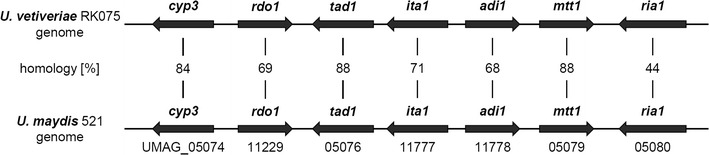
Itaconate clusters. Comparison of the itaconate cluster of *U.* *maydis* MB215 to the itaconate cluster of *U.* *vetiveriae* RK075 on protein level. Genes present in the itaconate cluster encode a putative Cytochrome P450 monooxygenase (*cyp3*), a putative ring-cleaving dioxygenase, a trans-aconitate decarboxylase (*tad1*), a Major Facilitator Superfamily transporter (*ita1*), an aconitate-Δ-isomerase (*adi1*), a putative mitochondrial tricarboxylate transporter (*mtt1*), and a putative transcriptional regulator (*ria1*). *Numbers* indicate NCBI BLAST identity in percentage

Since the clustered genes, responsible for itaconate production in *U.* *maydis* have been discovered recently [27], and the genome for *U.* *vetiveriae* was sequenced [55], we determined the presence of the itaconate cluster in this novel strain. Overall, proteins encoded in the *U.* *vetiveriae* cluster have 70–90% sequence similarity to their counterparts from *U.* *maydis* except for Ria1, which shows only 44% sequence identity (Fig. 3). This indicates that itaconate production likely proceeds via the same pathway [27]. This similarity is further supported, by the presence of 2-hydroxyparaconate in *U.* *vetiveriae* cultures, which is assumed to be a degradation product of itaconate [28], and the existence of the respective genes in the cluster.

### Medium optimization elevates itaconate production with *U.* *vetiveriae* TZ1

Even though production values both for malic acid, as well as itaconic acid were improved by ALE, the reached titers and production rates are still low compared to published values [16, 28, 40, 48, 53]. To further improve production properties, medium optimization was performed. The influence of differing concentrations of medium components on microbial production processes for organic acids, biomass, and proteins has been shown consistently in the literature for different organisms, such as *Aspergilli* [14, 56–58], Ustilaginaceae [21, 59], and *Corynebacterium* *glutamicum* [60]. Additionally, the used concentration of nitrogen and the used nitrogen source itself (e.g., ammonium chloride, yeast extract, and peptone) drastically changed acid production in different organisms [16, 29, 40, 59]. Consequently, we tested changing concentrations of NH_4_Cl (0.8, 1.6, 3.2 g L^−1^), FeSO_4_ (3, 13, 53, 103 mg L^−1^), KH_2_PO_4_ (0.125, 0.25, 0.5, 1 g L^−1^), and MgSO_4_ (0.1, 0.2, 0.5 g L^−1^), while keeping the concentration of all other components in the MTM unaltered. Additionally, we used peptone (2 g L^−1^) or yeast extract (2.4, 4.8 g L^−1^) instead of ammonium chloride. These two complex medium components contain 12.47 and 8.54% nitrogen, respectively, as determined by elemental analysis. Thus, the nitrogen (N) content of the different nitrogen sources was determined to correspond to 19 mM (0.8 g L^−1^ NH_4_Cl), 37 mM (1.6 g L^−1^ NH_4_Cl), 75 mM (3.2 g L^−1^ NH_4_Cl), 18 mM (2.0 g L^−1^ peptone), 15 mM (2.4 g L^−1^ yeast extract), and 0.29 mM (4.8 g L^−1^ yeast extract).

**Fig. 4:**
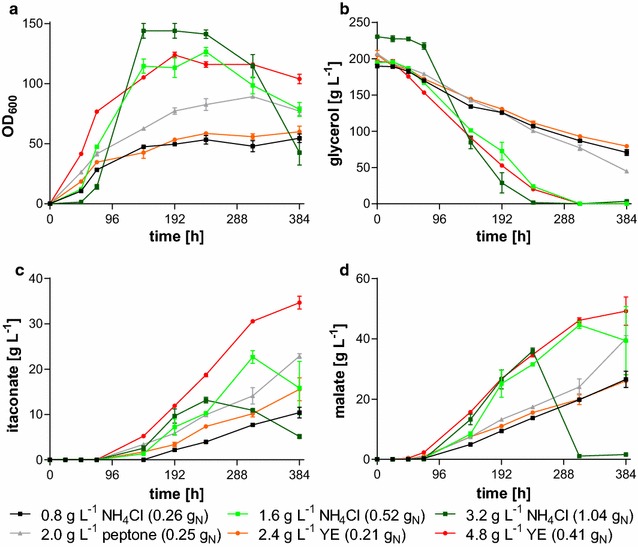
Medium optimization for *U.* *vetiveriae* TZ1. **a** OD_600_, **b** glycerol concentration, **c** itaconate concentration, and **d** malate concentration for *U.* *vetiveriae* TZ1 cultivated in 24-deep well plates containing MTM with 200 g L^−1^ glycerol, 100 g L^−1^ CaCO_3_ and different concentrations of NH_4_Cl, yeast extract (YE) or peptone. *Error bars* indicate deviation from the mean (*n* = 3)

Altered concentrations of FeSO_4_, KH_2_PO_4_, and MgSO_4_ did not change organic acid production with *U.* *vetiveriae* TZ1 (data not shown). Changing the nitrogen concentration and source itself, however, drastically improved growth, glycerol uptake, and organic acid production (Fig. 4).

As expected, a higher ammonium chloride concentration increased final OD_600_ and volumetric glycerol uptake rates. However, growth of *U.* *vetiveriae* was delayed, possibly due to higher stress levels resulting from high ammonium concentrations. This effect was previously observed using *U.* *trichophora* [41]. As expected, volumetric acid production rates improved with increasing NH_4_Cl concentrations, due to higher biomass concentrations. However, with 3.2 g L^−1^ NH_4_Cl, the final titer was reduced in comparison to 1.6 g L^−1^ NH_4_Cl. Even though in comparison to 0.8 g L^−1^ NH_4_Cl, the amount of supplied nitrogen was slightly lower for 2 g L^−1^ peptone, and 2.4 g L^−1^ yeast extract, itaconate production was greatly improved with the latter two N sources. In contrast to ammonium chloride, the use of these complex nitrogen sources resulted in an earlier onset of growth, consequently also resulting in an earlier production phase. Likely, yeast extract and peptone are less toxic to the cells in the initial growth stage, and their uptake and incorporation into biomass are energetically favorable. The uptake of di-, tri-, and possibly even oligopeptides is more efficient, since energy is spent for the uptake of one molecule, while several amino acids can be scavenged. The resulting surplus of energy leads to an overall improved biomass yield [61]. The highest itaconate titer of 34.7 ± 2.5 g L^−1^ was reached with 4.8 g L^−1^ yeast extract produced at a rate of 0.09 ± 0.01 g L^−1^ h^−1^. Simultaneously 46.2 ± 1.4 g L^−1^ malate was produced.

### Product inhibition by itaconate is likely stronger than product inhibition by malate

**Fig. 5:**
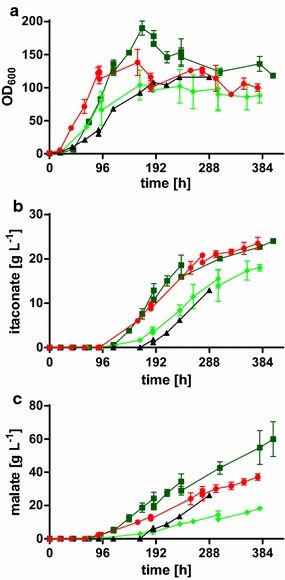
Controlled batch cultivations of *U.* *vetiveriae* TZ1 with different nitrogen concentrations. **a** OD_600_, **b** itaconate concentration, and **c** malate concentration, for cultures in MTM containing 200 g L^−1^ initial glycerol at 30 °C and pH 6.5 with DO kept at 80%. *Colors* indicate different initial nitrogen concentrations: 5 g L^−1^ yeast extract (*circles*, *red*), 1.6 g L^−1^ NH_4_Cl (*diamonds*, *light green*), 3.2 g L^−1^ NH_4_Cl (*squares*, *dark green*), and 6.4 g L^−1^ NH_4_Cl with doubled concentration of all medium components except glycerol (*triangles*, *black*). *Error bars* indicate deviation from the mean (*n* = 2)

For malate production with *U.* *trichophora* TZ1, a drastic increase in production rate could be achieved in controlled bioreactors [41]. Hence, we also investigated itaconate production with *U.* *vetiveriae* TZ1 in fed-batch cultivations with 200 g L^−1^ initial glycerol. Using 3.2 g L^−1^ NH_4_Cl or 5 g L^−1^ yeast extract resulted in a production rate of 0.06 ± 0.00 g L^−1^ h^−1^, which is similar to the values observed in 24-deep well plates (Fig. 5b; Table 2). Surprisingly, the titer (about 24 g L^−1^), was not increased for either culture, even though additional glycerol was fed throughout the fermentation. The experiments were repeated and the parameters were changed but in all bioreactor cultivations a titer of about 24 g L^−1^ itaconate could not be exceeded (data not shown). In CaCO_3_-buffered shake flasks, in contrast, higher concentrations were reached. This hints at product inhibition by itaconic acid concentrations above 24 g L^−1^.

**Table 2:** Characteristics of the bioconversion

	Titer (g L^−1^) (itaconate)	Rate (g L^−1^ h^−1^) (itaconate)	Yield (g_ita_ g_gly_^−1^) (itaconate)	Titer (g L^−1^) (malate)
5 g L^−1^ YE	23.5 ± 1.4	0.06 ± 0.00	0.08 ± 0.02	37.2 ± 2.0
1.6 g L^−1^ NH_4_Cl	18.0 ± 0.7	0.05 ± 0.00	0.06 ± 0.01	18.3 ± 1.3
3.2 g L^−1^ NH_4_Cl	24.1 ± 0.2	0.06 ± 0.00	0.07 ± 0.00	60.0 ± 10.4
6.4 g L^−1^ NH_4_Cl	13.3 ± 0.5	0.05 ± 0.00	0.07 ± 0.00	26.5 ± 0.4
4.8 g L^−1^ YE (shake flask)	34.7 ± 2.5	0.09 ± 0.01	0.18 ± 0.01	46.2 ± 1.4

Comparison of titer, rate, and yield for itaconate and titer of the main by-product malate for different bioreactor cultivations and the best shake flask cultivation. ±values indicate deviation from the mean (*n* = 3 for shake flask, *n* = 2 for bioreactors)

We described this effect previously in the context of malic acid formation with *U.* *trichophora* TZ1, where in CaCO_3_-buffered shake flasks and bioreactors, a concentration of about 200 g L^−1^ was reached, while in NaOH-buffered bioreactors a concentration of about 140 g L^−1^ was limiting [41]. The concentration of dissolved malic acid in the culture broth of CaCO_3_-buffered cultures was determined to be about 15 g L^−1^ throughout the cultivation, consequently lowering the osmotic stress for the cells [40]. For itaconic acid, the concentration of dissolved acid in CaCO_3_-buffered water was determined to be about 11 g L^−1^ (Tim Massmann, personal communication) with precipitation of the residual itaconate as calcium itaconate, strengthening the hypothesis of product inhibition by higher dissolved itaconic acid concentrations in NaOH-titrated bioreactors. Since also for *U.* *vetiveriae* TZ1 malic acid concentrations of up to 60.0 ± 10.4 g L^−1^ were reached in bioreactors, product inhibition by itaconic acid seems to be stronger than by malic acid. Also, a synergistic effect of inhibition by malate and itaconate cannot be excluded. Consequently, for a feasible production process, in situ product removal would be needed and the amount of malate as a by-product must be reduced. The possibility of in situ product removal for itaconate production has been demonstrated, making continuous production processes with Ustilaginaceae a promising option [34, 62]. Another possibility would be further ALE on higher itaconic acid concentrations in the medium, to obtain a strain which is less sensitive towards this product, provided that a loss of production can be avoided.

Apart from the observed product inhibition, the nitrogen source had a strong influence on fermentation performance. As expected, a lowered NH_4_Cl concentration (1.6 g L^−1^ NH_4_Cl) resulted in a lowered volumetric itaconate and malate production rate (Fig. 5b; Table 2), due to lower biomass formation (Fig. 5a). In contrast to results observed with *U.* *trichophora* TZ1 for malate production [41], 6.4 g L^−1^ NH_4_Cl combined with a doubled concentration for all other medium components resulted in neither rate nor titer improvement, rather reducing the growth rate and final OD_600_-values (Fig. 5a). In all, it seems that *U.* *vetiveriae* TZ1 is less tolerant to higher concentrations of ammonium or other medium salts than *U.* *trichophora* TZ1. In order to achieve higher cell densities and thus production rates, strains with improved tolerance towards higher salt concentrations can likely be isolated by additional ALE selection under ammonium stress, or an ammonium-fed process could be applied.

The use of 5 g L^−1^ yeast extract resulted in the same production values as the use of 3.2 g L^−1^ NH_4_Cl (Table 2), even though only 40% of the nitrogen is supplied (30 mM vs. 75 mM). Additionally, cultures grown in bioreactors with yeast extract showed an earlier onset of the growth and production phase, just as in shake flasks. However, the use of yeast extract in the production of bulk fermentation products is often a cost-prohibitive factor. Likely even with a higher concentration of NH_4_Cl or another nitrogen source, such as (NH_4_)SO_4_ of (NH_4_)NO_3_, the overall process would be more cost-effective. Cultivations with *U.* *maydis* using these nitrogen sources resulted in high acid titers compared to acidic nitrogen sources, such as NH_4_H_2_PO_4_ or NH_4_Cl, even though the main effect was argued to result from higher final pH values in barely buffered shake flask cultivations [20]. Yet, these observations would also correspond to first results with *U.* *vetiveriae* TZ1 cultivated in bioreactors at pH 4.5 and 5.5 (data not shown). At pH 4.5, no itaconate and malate production could be observed, while at pH 5.5 itaconate was still produced at a low titer of 8.0 ± 0.8 g L^−1^. Notably, in this cultivation, no malate was produced, suggesting a strategy for single product formation.

Even though itaconate production could not be improved in bioreactors, malate production was elevated. With 3.2 g L^−1^ NH_4_Cl, the malate titer increased to 60.0 ± 10.4 g L^−1^ produced within 403 h (Fig. 5c; Table 2). Just as for itaconate, malate production was reduced both with higher and lower NH_4_Cl concentrations. With yeast extract, malic acid production was improved compared to the production with 1.6 g L^−1^ NH_4_Cl, even though the contained nitrogen content is about 20% lower. These high values for malic acid underline the higher tolerance of Ustilaginaceae against malic acid compared to itaconic acid, even though, a specific production process for itaconic acid without by-product formation would be preferred.

### Metabolic engineering shifts organic acid production towards itaconate

Product specificity and hence product yield on substrate are important factors in microbial production processes. The simultaneous production of several organic acids in one strain results in a lowered titer for the desired product. Additionally, product recovery is more complex with similar compounds in the medium [63]. Thus, a strain producing one organic acid with high specificity is desirable. The possibility to improve microbial organic acid production processes by overexpression of the specific underlying production pathways has been shown consistently for different organisms and products [52, 53, 64–67]. In previous studies on itaconate production from glucose with *U.* *maydis* MB215, overexpression of the mitochondrial transporter *mtt1* and the regulator *ria1* of the itaconate gene cluster led to improved itaconate and reduced malic acid production [27, 68]. Additionally, the formation of the assumed degradation product of itaconate, 2-hydroxyparaconate, was influenced in the same way as itaconate production itself [28, 68]. Thus, in order to investigate whether it is possible to shift the product spectrum of *U. vetiveriae* towards itaconate in a similar manner, we created mutants of *U.* *vetiveriae* RK075 overexpressing either *mtt1* or *ria1* from *U.* *maydis* MB215. For overexpression, we used a plasmid (pUMa43 Otef–gfp–nos–cbx) for *U.* *maydis*, which confers resistance to carboxin by site-specific integration into the *ip* ^R^-locus. Previously we showed that this plasmid can also confer resistance to carboxin in other Ustilaginaceae, such as *U.* *trichophora*, even though site specificity is not given. Additionally, all contained genetic elements, such as promoter and terminator, were functional in other Ustilaginaceae [52].

**Fig. 6:**
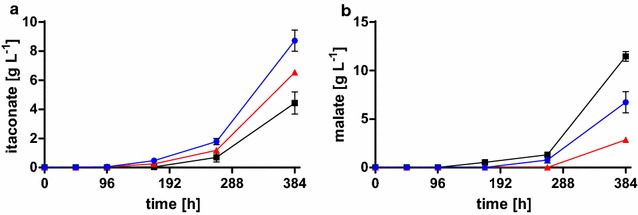
Overexpression of *ria1* and *mtt1* in *U.* *vetiveriae* RK075. **a** Itaconate and **b** malate concentration after 384 h cultivation for *U.* *vetiveriae* RK075 overexpressing *mtt1* (*red*), *ria1* (*blue*), and *U. vetiveriae* RK075 (*black*) cultivated in 24-deep well plates containing MTM with 200 g L^−1^ glycerol, 100 g L^−1^ CaCO_3_, and 0.8 g L^−1^ NH_4_Cl. *Error bars* indicate deviation from the mean (*n* = 3)

Cultivation of the *U.* *vetiveriae* overexpression mutants in 24-deep well plates containing MTM with 0.8 g L^−1^ NH_4_Cl, 200 g L^−1^ glycerol, and 100 g L^−1^ CaCO_3_ resulted in a 1.5-fold and twofold increased itaconate production after 384 h for *U.* *vetiveriae* overexpressing *mtt1* and *ria1*, respectively (Fig. 6a). Simultaneously, malate production was reduced to 75% for *mtt1* and 59% for *ria1* (Fig. 6b). Also the values for 2-hydroxyparaconate production were in line with the previously published results. This opens up further steps for improvement by deletion of the respective genes, since 2-hydroxyparaconate is an assumed degradation product of itaconate [28, 68].

This shift of organic acid production in favor of itaconate upon overexpression of either *ria1* or *mtt1* is comparable to the one in *U.* *maydis* MB215 both on glucose [68] and glycerol (data not shown). From these results, it was assumed that the mitochondrial transporter Mtt1 is the bottleneck of itaconate production in *U.* *maydis*, which can be overcome directly by overexpression of *mtt1*, or indirectly by overexpression of the regulator *ria1* [68]. This bottleneck seems also to be present in *U.* *vetiveriae* and can be overcome by single overexpression of *mtt1* or *ria1* from *U.* *maydis* MB215. These data indicate that not only the above-mentioned 70–90% sequence similarity for the proteins in the itaconate cluster of *U.* *vetiveriae* and *U.* *maydis* are sufficient for efficient heterologous expression but also the regulator of the itaconate cluster from *U.* *maydis* (*ria1*), in spite of a low similarity of 44%, is functional in *U.* *vetiveriae* and even other *Ustilago* strains, such as *U.* *xerochloae* (data not shown). In all, we were able to present *U.* *vetiveriae* TZ1 as promising production organism for itaconic acid from glycerol. First metabolic engineering attempts revealed the possibility to improve the product specificity by up-regulation of itaconate production from glycerol.

## Conclusions

Valorization of glycerol from biodiesel production has been a research focus for many years. The identified and improved *U.* *vetiveriae* strain TZ1 contributes a novel strategy, since it is able to produce high titers of organic acids from glycerol. Concentrations above 25 g L^−1^ itaconate seem to be inhibiting, consequently lowering the reached titers in NaOH-titrated bioreactors compared to CaCO_3_-buffered shake flasks, in which the product precipitates as calcium salt. The use of CaCO_3_ as buffering agent in bioreactors might help to overcome this limitation. Additionally, single-gene metabolic engineering allowed a reduction in the main by-product malate, thereby significantly increasing product specificity. Multi gene target metabolic engineering in the evolved *U. vetiveriae* TZ1 harbors huge potential to further improve strain performance. The here described workflow from primary screening, ALE, and medium optimization all the way to first metabolic engineering allows a rapid evaluation of novel host strains for the production of valuable products from alternative carbon sources.

## Methods

### Strains and culture conditions

The 76 strains belonging to the family Ustilaginaceae screened by Zambanini et al. [40] plus 50 additional strains were screened in this study (Table 3).

**Table 3:** Screened strains from the family Ustilaginaceae with final glycerol concentration

Name	Origin	Gly (g L^−1^)	Ita (g L^−1^)	Mal (g L^−1^)	Suc (g L^−1^)
*Cintractia axicola* BRIP 26922a	Queensland Plant Pathology Herbarium, Australia	55			
*S. modestum* BRIP 26928a	Queensland Plant Pathology Herbarium, Australia	51			
*C. lipocarphae* BRIP 26925a	Queensland Plant Pathology Herbarium, Australia	51			
*U. porosa* BRIP 26920a	Queensland Plant Pathology Herbarium, Australia	45			
*U. maydis* RK 212	[70]	42			
*U. lituana* BRIP 46795a	Queensland Plant Pathology Herbarium, Australia	42			
*U. avenae*	Centraalbureau Voor Schimmelcultures 131466	41			
*U. maydis* Nr. 484	American Type Culture Collection 22903	40			
*S. caledonicum* BRIP 28043a	Queensland Plant Pathology Herbarium, Australia	40			
*U. maydis* DSM 14603	Deutsche Sammlung von Mikroorganismen und Zellkulturen (DSMZ)	40			
*U. maydis* Nr. 213	Prof. M. Bölker, Philipps University Marburg, Germany	39			
*U. maydis* DSM 3121	Deutsche Sammlung von Mikroorganismen und Zellkulturen (DSMZ)	37			
*U. maydis* Nr. 477 a1bP	American Type Culture Collection 22895	34			
*U. maydis* Nr. 488	American Type Culture Collection 22907	34			
*U. cynodontis* NBRC 7530	NITE Biological Resource Center	34			
*U. maydis* Nr. 466 a1bE	American Type Culture Collection 22885	33			
*U. maydis* Nr. 483	American Type Culture Collection 22902	33			
*U. maydis* Nr. 197	Prof. M. Bölker, Philipps University Marburg, Germany	32			
*U. rabenhorstiana* NBRC 8995	NITE Biological Resource Center	31			
*U. maydis* Nr. 465 a1bD	American Type Culture Collection 22884	30			
*U. maydis* Nr. 206	Prof. M. Bölker, Philipps University Marburg, Germany	30			
*U. maydis* Nr. 467 a1bF	American Type Culture Collection 22886	30			
*U. maydis* Nr. 482	American Type Culture Collection 22901	29			
*U. cynodontis* NBRC 9727	NITE Biological Resource Center	29			
*U. maydis* Nr. 204	Prof. M. Bölker, Philipps University Marburg, Germany	29			
*U. maydis* Nr. 462 a1bA	Prof. M. Bölker, Philipps University Marburg, Germany	29			
*U. filiformis* UMa701	Centraalbureau Voor Schimmelcultures 131469	29			
*U. maydis* FB1 a1b1	Banuett & Herskowitz, 1989, Minnesota USA	28			
*U. maydis* RK 134	[70]	28			
*U. maydis* Nr. 485	American Type Culture Collection 22904	28			
*S. tumiforme* BRIP 26919a	Queensland Plant Pathology Herbarium, Australia	28	0.9	0.0	0.0
*U. maydis* RK 215	[70]	27			
*U. maydis* Nr. 489	American Type Culture Collection 22908	26			
*U. maydis* Nr. 470 a1bI	Prof. M. Bölker, Philipps University Marburg, Germany	25			
*U. maydis* RK 123	[70]	25			
*U. maydis* Nr. 198	Prof. M. Bölker, Philipps University Marburg, Germany	25			
*U. maydis* Nr. 207	Prof. M. Bölker, Philipps University Marburg, Germany	24			
*S. cruentum* UMa920 MAT1	Centraalbureau Voor Schimmelcultures 133249	24			
*U. cynodontis* NBRC 9758	NITE Biological Resource Center	23			
*U. maydis* RK 122	[70]	23			
*U. maydis* Nr. 490	American Type Culture Collection 22909	23			
*U. hordei* Uh4875-4 Mat1	[71]	23			
*U. maydis* Nr. 200	Prof. M. Bölker, Philipps University Marburg, Germany	22			
*U. maydis* Nr. 208	Prof. M. Bölker, Philipps University Marburg, Germany	22			
*U. maydis* Nr. 487	American Type Culture Collection 22906	21			
*U. maydis* Nr. 195	Prof. M. Bölker, Philipps University Marburg, Germany	21			
*U. maydis* MB215	Deutsche Sammlung von Mikroorganismen und Zellkulturen (DSMZ) DSM 17144	20			
*U. maydis* Nr. 209	Prof. M. Bölker, Philipps University Marburg, Germany	20			
*U. maydis* RK 213	[70]	20			
*S. consanguineum*	Centraalbureau Voor Schimmelcultures 131456	19			
*U. maydis* Nr. 478 a1bQ	American Type Culture Collection 22896	19			
*U. maydis* RK 214	[70]	19			
*U. maydis* Nr. 212	Prof. M. Bölker, Philipps University Marburg, Germany	19			
*U. maydis* Nr. 215	Prof. M. Bölker, Philipps University Marburg, Germany	19			
*U. maydis* Nr. 214	Prof. M. Bölker, Philipps University Marburg, Germany	18			
*U. maydis* Nr. 205	Prof. M. Bölker, Philipps University Marburg, Germany	18			
*U. maydis* Nr. 463 a1bB	American Type Culture Collection 22882	18			
*U. maydis* Nr. 481	American Type Culture Collection 22900	18			
*U. maydis* Nr. 196	Prof. M. Bölker, Philipps University Marburg, Germany	17			
*U. maydis* Nr. 199	Prof. M. Bölker, Philipps University Marburg, Germany	17			
*U. maydis* Nr. 492	American Type Culture Collection 22911	17			
*U. maydis* Nr. 201	Prof. M. Bölker, Philipps University Marburg, Germany	17			
*U. maydis* RK 139	[70]	16			
*C. lipocarphae* BRIP 26927a	Queensland Plant Pathology Herbarium, Australia	15			
*U. maydis* Nr. 469 a1bH	American Type Culture Collection 18604	14			
*U. maydis* Nr. 479 a1bR	American Type Culture Collection 22897	14			
*U. maydis* Nr. 211	Prof. M. Bölker, Philipps University Marburg, Germany	14			
*S. exsertum* RK 033	Centraalbureau Voor Schimmelcultures 131457	14			
*U. schmidtiae* BRIP 26906a	Queensland Plant Pathology Herbarium, Australia	13			
*U. maydis* Nr. 491	American Type Culture Collection 22910	13			
*Ustanciosporium gigantosporum* UMa706	Centraalbureau Voor Schimmelcultures 131478	12			
*Cintractia sp*. BRIP 60413a	Queensland Plant Pathology Herbarium, Australia	12			
*U. maydis* Nr. 480	American Type Culture Collection 22899	12			
*U. maydis* Nr. 495	American Type Culture Collection 221914	12			
*U. maydis* DSM 4500	Deutsche Sammlung von Mikroorganismen und Zellkulturen (DSMZ)	11			
*U. maydis* HB1990	Biotechnology And Information Research Network AG, Zwingenberg, Germany	11			
*U. maydis* Nr. 476 a1bO	American Type Culture Collection 22894	11			
*S. setariae* BRIP 26910a	Queensland Plant Pathology Herbarium, Australia	10			
*C. sp*. BRIP 60422a	Queensland Plant Pathology Herbarium, Australia	10			
*U. maydis* Nr. 202	Prof. M. Bölker, Philipps University Marburg, Germany	8			
*M. spermophorus* BRIP 60430a	Queensland Plant Pathology Herbarium, Australia	7			
*M. spermophorus* BRIP 60448a	Queensland Plant Pathology Herbarium, Australia	7			
*S. scitamineum* UMa698, Sscl4, JS109, MAT1	Centraalbureau Voor Schimmelcultures 131462	7			
*S. ovarium* BRIP 26909a	Queensland Plant Pathology Herbarium, Australia	6			
*S. themedae* BRIP 26917a	Queensland Plant Pathology Herbarium, Australia	6			
*S. aristidicola* BRIP 26930a	Queensland Plant Pathology Herbarium, Australia	6			
*U. maydis* Nr. 471 a1bJ	American Type Culture Collection 22889	5			
*U. cynodontis* BRIP 28040a	Queensland Plant Pathology Herbarium, Australia	5			
*U. maydis* Nr. 203	Prof. M. Bölker, Philipps University Marburg, Germany	4			
*S. walkeri* RK 031	Centraalbureau Voor Schimmelcultures 131464	4			
*A. heteropogonicola* BRIP 60896a	Queensland Plant Pathology Herbarium, Australia	3			
*C. mitchellii* BRIP 26923a	Queensland Plant Pathology Herbarium, Australia	2			
*U. maydis* FB2 a2b2	Banuett & Herskowitz, 1989, Minnesota USA	2			
*Anthracocystis sehimatis* BRIP 60890a	Queensland Plant Pathology Herbarium, Australia	2	0.0	0.0	0.0
*U. maydis* Nr. 474 a1bM	American Type Culture Collection 22892	2			
*P. antarctica* NBRC 10260	NITE Biological Resource Center	1			
*A. bothriochloae* BRIP 60901a	Queensland Plant Pathology Herbarium, Australia	1	0.0	0.0	0.0
*U. cynodontis* UMa709	Centraalbureau Voor Schimmelcultures 131467	1			
*S. iseilematis*-*ciliati* BRIP 60429a	Queensland Plant Pathology Herbarium, Australia	0			
*M. ordensis* BRIP 26904a	Queensland Plant Pathology Herbarium, Australia	0	1.0	0.0	0.0
*U. curta* BRIP 26929a	Queensland Plant Pathology Herbarium, Australia	0			
*S. lanigeri* BRIP 27609a	Queensland Plant Pathology Herbarium, Australia	0	1.0	0.0	0.0
*U. maydis* Nr. 473 a1bL	American Type Culture Collection 22891	0			
*M. eriachnes* RK 028	Centraalbureau Voor Schimmelcultures 131454	0			
*U. trichophora* RK089	Centraalbureau Voor Schimmelcultures 131473	0			
*U. vetiveriae* RK 075	Centraalbureau Voor Schimmelcultures 131474	0	0.6	0.7	0.2
*U. xerochloae* UMa702	Centraalbureau Voor Schimmelcultures 131476	0	0.2	1.7	0.0
*P. hubeiensis* NBRC 105053	NITE Biological Resource Center	0	1.0	0.0	0.0
*P. hubeiensis* NBRC 105054	NITE Biological Resource Center	0	0.7	0.0	0.0
*P. hubeiensis* NBRC 105055	NITE Biological Resource Center	0	0.7	0.0	0.0
*U. trichophora* NBRC 100155	NITE Biological Resource Center	0	2.2	0.0	0.7
*U. trichophora* NBRC 100156	NITE Biological Resource Center	0	1.8	0.0	0.7
*U. trichophora* NBRC 100157	NITE Biological Resource Center	0	1.5	0.0	0.5
*U. trichophora* NBRC 100158	NITE Biological Resource Center	0	0.0	0.0	0.0
*U. trichophora* NBRC 100159	NITE Biological Resource Center	0	1.4	0.0	0.4
*U. trichophora* NBRC 100160	NITE Biological Resource Center	0	2.1	0.0	0.3
*P. tsukubaensis* NBRC 1940	NITE Biological Resource Center	0	0.8	0.1	0.0
*M. mackinlayi* BRIP 52549a	Queensland Plant Pathology Herbarium, Australia	0	1.1	0.0	0.0
*S. cenchri*-*elymoidis* BRIP 26491a	Queensland Plant Pathology Herbarium, Australia	0	1.7	0.0	0.0
*S. bothriochloae* BRIP 26908a	Queensland Plant Pathology Herbarium, Australia	0			
*U. triodiae* BRIP 26907a	Queensland Plant Pathology Herbarium, Australia	0			
*M. tubiformis* BRIP 60434a	Queensland Plant Pathology Herbarium, Australia	0	0.0	0.0	0.0
*U. xerochloae* BRIP 60876a	Queensland Plant Pathology Herbarium, Australia	0	2.7	0.0	0.2
*S. iseilematis*-*ciliati* BRIP 60887a	Queensland Plant Pathology Herbarium, Australia	0	0.0	2.5	0.0
*A. caledonica* BRIP 60892a	Queensland Plant Pathology Herbarium, Australia	0	0.0	0.0	0.0
*U. egenula* BRIP 60884 a	Queensland Plant Pathology Herbarium, Australia	0	0.0	0.0	0.0

As standard medium, MTM was used according to Zambanini et al. containing 100 g L^−1^ CaCO_3_ with differing concentrations of FeSO_4_, MgSO_4_, and KH_2_PO_4_ and differing concentrations of NH_4_Cl, yeast extract, or peptone (see text for details) [40].

Adaptive laboratory evolution (for 62 days), medium optimization, preparation of pre-cultures, shake flask experiments, and batch cultivations were conducted as described previously [40, 41]. For batch cultivations, the pH was set to 6.5 and controlled by automatic addition of 10 M NaOH.

### Analytical methods

All experiments were performed in duplicates. Shown is the arithmetic mean of the duplicates. Error bars and ± values indicate deviation from the mean.

Samples were treated as described previously [40, 41]. OD_600_ determination and HPLC analysis were performed as described previously [40]. Ammonium concentration was determined by a colorimetric assay according to Willis [69].

The nitrogen content of peptone and yeast extract was determined by Mikroanalytisches Laboratorium Kolbe_(Nachf.)_ (Mülheim an der Ruhr, Germany).

### Cloning procedures

For overexpression of *ria1* and *mtt1*, the overexpression constructs generated by Geiser et al. were used [27].

All cloning procedures were performed as described previously [52].
